# Breast Cancer and Arsenic Anticancer Effects: Systematic Review of the Experimental Data from *In Vitro* Studies

**DOI:** 10.1155/2022/8030931

**Published:** 2022-12-29

**Authors:** Anna Skoczynska, Marta Skoczynska

**Affiliations:** ^1^Wrocław Medical University. Department of Internal and Occupational Diseases, Hypertension and Clinical Oncology, Pasteura 1, 50-556 Wrocław, Poland; ^2^Lower Silesia Specialist Hospital, Department of Rheumatology and Internal Diseases, Fieldorfa 2, 54-049 Wrocław, Poland

## Abstract

Arsenic is a known environmental carcinogenic agent. However, under certain circumstances, it may exert anticancer effects. In this systematic review, we aim to provide information on recent developments in studies on arsenic antitumor effects in breast cancer. Research included in the review refers to experimental data from *in vitro* studies. The data was collected using search terms “breast cancer,” “arsenic,” and “anticancer” (25.05.2021). Only studies in English and published in the last 10 years were included. The search identified 123 studies from the EBSCOhost, PubMed, and Scopus databases. In the selection process, thirty full-texts were evaluated as eligible for the review. The literature of the last decade provides a lot of information on mechanisms behind anticancer effects of arsenic on breast cancer. Similar to arsenic-induced carcinogenesis, these mechanisms include the activation of the redox system and the increased production of free radicals. Targets of arsenic action are systems of cell membranes, mitochondria, pathways of intracellular transmission, and the genetic apparatus of the cell. Beneficial effects of arsenic use are possible due to significant metabolic differences between cancer and healthy cells. Further efforts are needed in order to establish modes and doses of treatment with arsenic that would provide anticancer activity with minimal toxicity.

## 1. Introduction

Arsenic, a metalloid found in organic and nonorganic compounds, is an environmental factor affecting human health mainly due to its presence in drinking water and in the air [1, 2]. Air contamination with arsenic is anthropogenic and concerns areas affected by mining and copper smelting [2]. Arsenic contained in the drinking water is of a natural origin and in some locations present in the groundwater. About 140 million people worldwide were reported to be drinking water containing arsenic at concentrations higher than the WHO acceptable guideline value of 10 *μ*g/L [3]. Food such as seafood and agricultural crops from arsenic-contaminated areas is another important source of arsenic [4].

Arsenic is a proven toxic and carcinogenic factor. Its toxicity is associated with inhibition of about 200 enzymes, including enzymes mediating cellular energy pathways as well as DNA synthesis and repair [5]. International Agency for Research on Cancer (IARC) has classified arsenic as a class I carcinogen [4]. The carcinogenic effects of arsenic concern the skin, lungs, the bladder, and the prostate [1]. Recently, increasing attention has been paid to the possible relationship between environmental exposure to arsenic and the incidence of breast cancer. The reason for this interest is the increasing incidence of breast tumors and search for new risk factors. Breast cancer is the most common cancer as well as the most frequent cause of cancer-related death among women worldwide [6]. Risk factors for this cancer include advanced age and mutations in *BRCA1* or *BRCA2* genes, as well as lifestyle, reproductive, and environmental factors such as diet, obesity, early menarche, late menopause, older age at first birth, smoking, excess alcohol drinking, other malignancies, and exposure to carcinogens such as arsenic [6–8].

**Figure 1:**
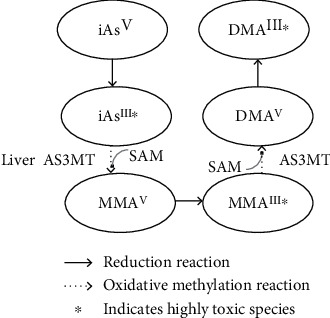
Inorganic arsenic metabolism pathway as proposed by Challenger, 1947. Methylation occurs using the arsenic-3-methyltransferase (AS3MT) enzyme, and methyl donor S-adenosylmethionine (SAM) [15]

However, data on the relationship between the occurrence of breast cancer and arsenic exposure has been inconsistent. In one ecological study, Hinwood et al. showed a positive association between airborne arsenic concentration and breast cancer incidence [8]. In other studies, arsenic exposure was associated with a two-fold increased risk for breast cancer [9], and the blood arsenic level was shown to be a potentially useful marker of cancer risk [10]. Moreover, a systematic review study from Khanjani et al. provided some evidence of a relationship between arsenic exposure and breast tumor in some groups of women [11]. However, other ecological studies failed to demonstrate a significant connection between arsenic exposure and incidence of breast cancer [12, 13]. It is possible that diverse results from various studies were due to varied human ability to methylate arsenic subspecies [14]. Ingested inorganic trivalent arsenite (iAs^III^) is methylated in hepatocytes to the first intermediate, monomethylarsonic acid (MMA^V^), which is then reduced to highly genotoxic and cytotoxic monomethylarsonous acid (MMA^III^) [15], Figure 1.

Women with an increased capacity to methylate inorganic arsenic compounds into MMA^III^ and a reduced capacity to perform further methylation from MMA^III^ to dimethylarsinous acid (DMA^III^) have a higher risk of developing breast cancer, regardless of genetic polymorphisms [16]. Polymorphisms of genes associated with arsenic metabolism seem to be important in breast cancer risk estimation. In one case-control study, polymorphism in MTR c.2756A > G protected against breast cancer associated with exposure to inorganic arsenic [17]. However, despite results from these studies, a recent meta-analysis did not confirm the causal link between arsenic exposure and breast cancer, perhaps due to the insufficient amount of data [7].

Interestingly, a variety of clinical and experimental studies indicated that inorganic arsenic and its methylated metabolites may have either carcinogenic or anticancer effects [18]. In isolated human breast cells, arsenic compounds can stimulate epigenetic disruption on many cellular processes, leading to carcinogenesis [15]. In animal studies, data on arsenic carcinogenicity remains scarce [19], although transplacental carcinogenicity of inorganic arsenic from the drinking water was shown in mice [20]. But, even with regard to breast cancer cells, the question of whether arsenic has always carcinogenic properties due to epigenetic disruption is not clear. Altered expression of cell cycle-related genes associated with DNA demethylation, associated with apoptosis and cycle arrest, seems to be one example of arsenic antitumor action [21]. Arsenic induces functional reexpression of estrogen receptor alpha by demethylation of DNA in estrogen receptor-negative human breast cancer [22]. Another anticancer mechanism of arsenic action may be through increased cellular antioxidant activity in mammary cancer cells, which may improve response to chemotherapy [23].

**Figure 2:**
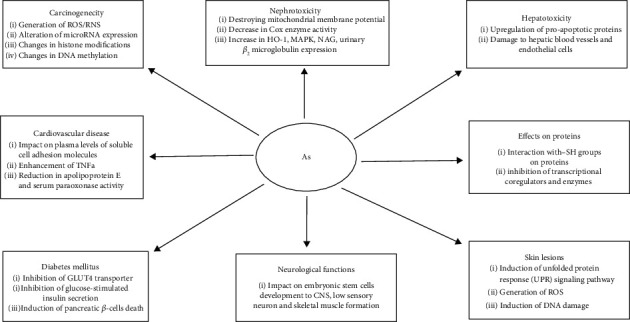
Pathological effects of arsenic on a cellular and molecular level [18].

Arsenic compounds such as arsenic trioxide (ATO) or arsenic hexoxide (AS6) have been used as cancer treatment for a long time. For example, ATO has been demonstrated to be an efficient drug in the therapy of acute promyelocytic leukemia (APL) [24, 25], whereas AS6 has been used in Korea as a folk treatment of cancer since the late 1980s [26]. APL is characterized by t(15; 17)(q22; q21) chromosomal translocation, resulting in the fusion of the promyelocytic leukemia (PML) gene with the retinoic acid receptor-*α* (RAR*α*) gene. The *PML/RARα* fusion gene encodes a protein blocking differentiation of hematopoietic progenitor cells in the bone marrow, leading to the development of APL [27–30]. Anticancer activity of ATO in APL results from ATO-induced PML/RAR*α* fusion protein degradation. Arsenite, a hydrolyzed form of ATO, specifically binds to cysteine residues of zinc finger motifs in PML B-box2 domain, inducing PML/RAR*α* fusion protein's structural and functional changes, leading to its degradation and deactivation [31, 32]. In recent years, the double induction therapy that is the combination of ATO with all-trans retinoic acid has become a standard regimen for APL treatment [30, 32]. However, although arsenic therapy has been successful in APL and other malignancies, dose-dependent arsenic toxicity has limited its clinical use [18, 30, 33–35]. Pathological effects of arsenic are presented in Figure 2 [18].

In studies performed *in vitro*, the extent of toxicity depended on type of animal models and cells used in experiments as well as on the rate of uptake of studied arsenic compounds [18]. Arsenic is a known oxidative stress inducer [36] and molecular mechanisms for both carcinogenic and anticancer effects of arsenic compounds seem to include their effect on the cellular redox system [37, 38]. The impact of the arsenic used at various, increasing doses on redox potential in breast cancer cell lines has been the subject of a growing interest in the last 10 years. The anticancer effect of arsenic is associated with the cell cycle arrest and apoptosis. The first action is controlled by cyclins and cyclin-dependent kinases [39]. Apoptosis is associated with redistribution of apoptosis-related proteins and activation of enzymes such as caspases or PARP [40, 41].

The molecular mechanisms of the anticancer effects of arsenic have been fragmentarily studied. The aim of the manuscript was to review available experimental studies on the anticancer activity of arsenic in breast cancer cells.

## 2. Methods

**Figure 3:**
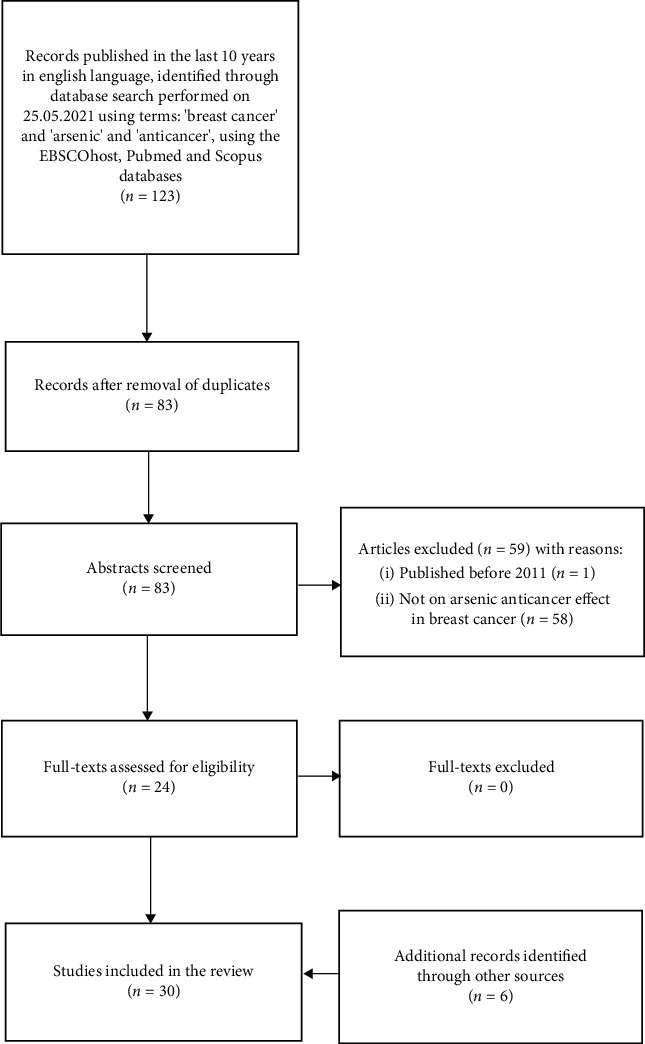
PRISMA chart.

We carried out a systematic search of the available literature published in the last 10 years, using search terms “breast cancer,” “arsenic,” and “anticancer.” Only experimental studies in English were included in the final analysis. The search was performed using the EBSCOhost, PubMed, and Scopus databases, and resulted in 123 publications. Forty results were removed as duplicates. Reviewing the remaining 83 abstracts led to the removal of one study published before 2011 as well as 58 studies not on anticancer effect of arsenic in breast cancer. The remaining 24 full-texts were evaluated as eligible for the review. Six studies identified through another source were added at a later date (Figure 3).

The selection process enabled a comprehensive review of recent experimental studies on arsenic antitumor properties in breast cancer and molecular mechanisms behind them. The main limitation of our review was not including studies in languages other than English or early studies on arsenic anticancer effects.

## 3. Results

The thirty studies identified through the systematic search are summarized in Table 1. Presented studies were performed with the use of human breast cancer cell lines, most often MCF-7 (Michigan Cancer Foundation 7). MCF-7 line is a mammary epithelial cancer cell line (ER+, PR±, and HER2-) which belongs to the luminal subtype of human breast cancer and is associated with low levels of invasion. Another frequently used line was MDA-MB-231 cell line (ER-, PR-, and HER2-), known also as triple negative breast cancer (TNBC) cell line, derived from highly invasive basal-B cancer subtype. Other lines such as MDA-MB-468 cells, which belong to the basal-A subtype and are associated with intermediate invasive activity; SK-BR-3 cells, that overexpress the Her2 gene product; ZR-75-1 cell lines, derived from human Caucasian breast carcinoma; and T-47D cells, human epithelial breast tumor cells, were used less frequently. In three studies, the anticancer activity of arsenic was evaluated also *in vivo* on mice xenograft model of human breast cancer [42–44]. In some studies, breast cancer cells were transfected with different types of nucleic acids, such as DNA plasmids or small noncoding RNAs.

The arsenic compound most frequently studied for its anticancer activity was arsenic trioxide. Also, arsenic hexoxide, arsenic disulfide, sodium arsenite, monomethylarsonous acid, or dimethylarsinous acid were used. In experiments performed *in vitro*, the doses of arsenic trioxide have been usually titrated, at the range of low doses, i.e., from 0.0 to 5.0 or 6.0 micromolar (*μ*M). The time of arsenic action was 24, 48, and 72 hours. However, ATO at high concentrations (up to 20 *μ*M) was also applied [45].

The anticancer effects of arsenic are evaluated on different levels of the intracellular structure and metabolism. In studies on breast cancer, ATO concentration of 1.0 or 2.0 *μ*M in studies *in vitro*, or ATO at a dose of 2 mg/kg 3 times per week in experiments on mice, or AS6 concentration of 5 *μ*M *in vitro* are recognized as clinically safe [26, 42]. Establishing a safe dose of arsenic compounds in clinical treatment of patients with breast cancer proved to be difficult. Thus, numerous *in vitro* experiments on combination therapy with arsenic and compounds with synergistic antitumor properties have been performed in order to allow for the reduction of the therapeutic dose of arsenic and the associated toxicity.

### 3.1. Combined Treatment Reducing Arsenic Toxicity

One such attempt concerned the combined use of arsenic and tetrandrine. Tetrandrine (Tetra) is an alkaloid isolated from the root of *Stephania tetrandra S*, acting as a calcium channel blocker which inhibits arsenic-induced cardiotoxicity. Yuan et al. studied the combined effect of sodium arsenite (NaAsO_2_, As^III^) and Tetra on the MDA-MB-231 cells in experiments *in vitro* and in studies *in vivo* on the nude mice. The MDA-MB-231 cell line has been chosen as this subtype of cancer is “the most difficult to treat due to its aggressive, metastatic behavior and lack of a targeted therapy” [43]. In response to As^III^ or Tetra alone, a dose-dependent decrease in MDA-MB-231 cells viability was observed, whereas combined exposure caused a synergistic effect.

The synergistic antitumor activity of As^III^ and Tetra was observed also in human breast cancer xenograft murine model. Importantly, the combined treatment with As^III^ and Tetra was shown to be well tolerated in mice. On the level of the cell cycle, an increase in the quantity of MDA-MB-231 cells in the S phase and a decrease in the G0/G1 phase was observed. These changes were enhanced by the combined treatment and associated with increased expression of FOXO3a transcription factor and p27 as well as decreased expression of cyclin D1. Furthermore, combined treatment with As^III^ and Tetra was associated with an increased LDH leakage and the upregulated expression of (a) LC3, an autophagic marker; (b) phosphorylated AMP-activated protein kinase (AMPK), important in terms of cellular energy homeostasis and autophagy induction, (c) total AMPK; and (d) beclin-1, playing a central role in autophagy. Results obtained in experiments *in vitro* were in general consistent with results obtained in studies performed *in vivo* [43].

The anticancer effects of ATO at clinically safe doses (1.0 or 2.0 *μ*M) in MDA-MB-231 cells can be synergistically enhanced also by additional treatment with ATRA (all-trans retinoic acid), a derivative of vitamin A [42]. In a study performed *in vivo* on both TNBC orthotopic xenografts and TNBC patient-derived tumor transplanted to mice, ATO used at a dose of 2 mg/kg 3 times per week in combination with ATRA markedly inhibited tumor growth by synergistic inhibition of multiple oncogenic pathways. One example was inhibition of tumor initiating cells (TICs). These cells, resistant to pharmacotherapy, are responsible not only for tumor growth but also for metastatic activity [42].

In another study, MCF-7 cells were exposed to three arsenic species: iAs^III^ and its metabolites, MMA^III^, and DMA^III^, given either with or without cryptotanshinone (CPT) [41]. CPT, a quinoid diterpene, isolated from the root of the Asian medicinal plant *Salvia miltiorrhiza*, has been identified to possess at subtoxic concentrations anti-inflammatory, anticancer, antioxidative, and antiangiogenic properties. The authors suggested that antiproliferative effects of CPT include inhibition of the mammalian target rapamycin (mTOR) signaling pathway and inhibition of signal transducer and activator of transcription 3 (Stat3) pathway activation, resulting in inhibition of cyclin D1 expression. Increased apoptosis was observed in MCF-7 cells simultaneously, exposed to low doses of MMA^III^ and CPT, while these cells were less sensitive to administration of MMA^III^ or DMA^III^ alone and resistant to iAs^III^ or CPT. The increased apoptosis was associated with redistribution of Bax and Bak, proapoptotic proteins, in the mitochondria, as well as activation of two enzymes: PARP and caspase-9 [41].

Aside from Tetra and CPT, another potential plant product enhancer of arsenic anticancer activity is cotylenin A [46]. Cotylenin A (CN-A) potentiated ATO-induced inhibition of cell growth. MCF-7 cell treated simultaneously with ATO and CN-A displayed growth inhibition due to synergistic induction of cleaved caspase-7 and inhibition of oxidative response and cell survival. Combined exposure to ATO and CN-A led also to the suppression of MDA-MB-231 cells invasive capacity [47].

Another biological compound used in breast cancer cotreatment studies is melatonin (N-acetyl-5-methoxytryptamine). In many types of cancer cells, melatonin exerts proapoptotic, antiproliferative, and antiangiogenic activities. Yun et al. showed the synergistic effect of melatonin and ATO in increasing apoptosis in breast cancer cells. This effect was associated with an increased expression of cleaved PARP (a marker of apoptosis) and an increased expression of Bax and a decreased expression of survivin and Bcl-2, antiapoptotic proteins. Apoptosis induced by cotreatment with melatonin and ATO was associated with increased ROS generation and upregulation of novel Redd1 gene. This gene is responsible for cell stress and hypoxia. In SK-BR-3 and MDA-MB-231 cells, the sustained upregulation of Redd1 induced apoptosis associated with activation of the p38 and the c-Jun N-terminal kinase (JNK) [48]. Increased expression of Bax, accompanying decreased expression of survivin and Bcl-2 resulted also from MCF-7 combined treatment with ATO, epigallocatechin-3-gallate (EGCG), and gamma radiation. EGCG, contained mainly in green tea, is a polyphenol with strong antioxidant and anticancer properties. The use of EGCG together with ATO showed an antiproliferative effect in MCF-7 cells, which was dose- and time-dependent [49]. When the therapy was extended to gamma radiotherapy, an up to 80% increase in cancer cell death rate was observed [49].

### 3.2. Association between Anticancer Effect of Arsenic and ROS Generation

Another mechanism for enhanced breast cancer cells death may be enhanced intracellular ROS generation due to treatment with ATO combined with inhibition of Flap Endonuclease 1 (FEN1) expression [44]. The study performed *in vitro* indicated that ATO suppressed MDA-MB-231 and MDA-MB-468 cells growth in an ATO dose- and time-dependent manner, triggered DNA damage and upregulated FEN1 expression. TNBC cells transfected with FEN1-specific siRNA, knockdown (KD) of FEN1, followed by treatment with ATO at different doses, displayed significantly impaired cells viability. Therefore, silencing FEN1 expression could increase the sensitivity of TNBC cells to arsenic. This conclusion was supported by the results of studies performed *in vivo* on mouse xenograft models. Apoptosis-related proteins expression measured in TNBC cells was enhanced by ATO at low doses (2.5 or 5.0 *μ*M) and this effect was reversed by N-acetylcysteine (NAC). Furthermore, while ATO and FEN1-KD alone were poor p38 and JNK pathway activators, ATO and FEN1-KD used together significantly increased phosphorylation of p38 and JNK. This effect was also reversed by NAC, indicating that increased ROS generation in TNBC cells could induce cell death through p38 and JNK pathway [44].

Aside from oxidative stress, also, nitrosative stress (NS) may be involved in arsenic anticancer effect. NS is a proposed anticancer mechanism of sodium arsenite (NaAsO_2_) [50]. Reactive nitrogen species arise mainly from nitric oxide, which has cytoprotective activity. In MCF-7 and ZR-75-1 cell lines, many linear correlations between membrane stress parameters and nitrites or reducing phenolic compounds were demonstrated. L-citruline formation was increased in arsenic-treated cells, to a greater effect in MCF-7 cells, whereas incubation with silymarin or quercetin lead to inhibition of L-citruline generation in both cell lines. On the other hand, ZR-75-1 cells showed a greater cytoplasmatic reducing activity than MCF-7 cells [50].

### 3.3. Arsenic and Mitochondrial Function

An increased generation of intracellular ROS by ATO plays a role also in mitochondrial dysfunction. One of known targets for ATO is mitochondrial transition pore (MTP). ATO may impact mitochondrial membrane potential (MMP), induce opening of MTP and increase ROS and cytochrome c release from mitochondria [45]. In breast cancer cells cotreatment with ATO (5-7 *μ*M) and dichloroacetate (DCA), one of pyruvate dehydrogenase kinase inhibitors which reverse the Warburg effect by redirecting ATP synthesis from glycolysis to oxidative phosphorylation, synergistically reduced cell proliferation, changed ATP level, and induced cell death. In T-47D line cells, DCA and ATO together decreased MMP depolarization and led to a reduction in expression of two major transcription factors responsible for the Warburg effect and mitochondrial activity regulation, i.e., c-Myc and HIF-1*α*. Two mechanisms on the level of mitochondrial metabolism that enhance anticancer effect of a combined treatment with ATO and DCA were described: the reversing of the Warburg effect by DCA and the inhibition of oxidative phosphorylation by ATO. Both mechanisms interfere with the energy homeostasis of cancer cells [45].

### 3.4. Posttranscriptional Effects of ATO

Antitumor effect of ATO on the posttranscriptional level is partially mediated by ATO-induced microRNAs inhibition. MicroRNAs, small, noncoding RNAs, depending on their targets, can be oncogenes or tumor suppressors. One of promising therapeutic targets is miR-27a. Anticancer ATO effects in breast cancer may be dependent on ATO-induced inhibition of miR-27a [51]. Inhibitory action of ATO is followed by upregulation of Fbw7 gene, leading to apoptosis, inhibition of cell proliferation, and invasion [51].

Another microRNA, let-7a, may have important clinical significance. The let-7a present in breast cancer cells displays anticancer effect and this effect may be enhanced by ATO [52]. It has also been shown that the upregulation of let-7a could promote anticancer effects of ATO. A potential target gene of let-7a is Notch-1 [52]. ATO may inhibit the Notch-1 [53].

In breast cancer cells, ATO may be included not only in Notch-1 but also in other oncogenic signaling pathways. Pin1 (peptidyl-prolyl cis/trans isomerase) is known as a critical regulator of signaling networks. Pin1 activates many oncoproteins, simultaneously inactivates many tumor suppressors and downregulates global microRNA. Kozono et al. showed that ATO inhibited and degraded Pin1 as well as suppressed its oncogenic function [42]. In TNBC cells and in animal models including patient-derived orthotopic xenografts expressing Pin1, use of ATO effectively reduced Pin1 levels and its substrate oncoproteins (nuclear factor kappa B-NF*κ*B, p65, *β*-catenin, and Rab2A). Simultaneously, arsenic increased levels of Pin1 substrate tumors suppressors such as Fbw7 [42]. Multiple analyses confirmed the synergism between ATO and ATRA, another Pin1 inhibitor, in anticancer effects. ATRA inhibited and degraded Pin1, and also induced aquaporin 9, which increased ATO cellular uptake [42].

### 3.5. Arsenic Anticancer Effect on Regulation of Apoptosis Pathway Gene Expression and Cell Cycle

One of the mechanisms for ATO-induced apoptosis and cell cycle arrest may be DNA hypomethylation. ATO has been identified as a DNA methylation inhibitor. Moghaddaskho et al. determined the genes group promoter methylation status in various lines of breast cancer cells. All genes were responsible for cell cycle regulation [21]. In breast cancer cells, only three genes, including cyclin D2, were abnormally hypermethylated, and ATO led to their demethylation. Furthermore, ATO increased mRNA expression of these genes. Authors hypothesized that while methylation of cell cycle genes led to their silencing, ATO increased their expression through demethylation [21]. An indicator of increased expression of cell cycle inhibitory genes may be increased levels of p21 and p27 proteins. Such changes were demonstrated in MCF-7 cells treated with ATO [54].

The enhanced sensitivity of MCF-7 cells to ATO at lower concentrations may result from the inhibition of phosphatidylinositol-3 kinases (PI3Ks). PI3Ks are important intracellular secondary messengers involved in many cellular processes. The signal pathway of PI3Ks and its nuclear effectors, in particular FOXO transcription factors, specify target gene expression important in cell cycle arrest, cell death, ROS detoxification, DNA repair, energy homeostasis, and glucose metabolism. The PI3K inhibitor (BKM120) augmented ATO-induced antiproliferative effects by inducing G1 arrest and reducing the incorporation of bromodeoxyuridine into the synthesized DNA [46]. This last action was associated with suppression of the human telomerase reverse transcriptase (hTERT) expression. Moreover, in the presence of the BKM120, ATO-induced apoptosis was increased [46].

Compared with ATO, arsenic disulfide (As_2_S_2_) is less toxic while it has similar anticancer effects [39]. In breast cancer, As_2_S_2_ at concentrations of 0-24 *μ*M inhibited cell viability in both a dose- and time-dependent manner, and MCF-7 cells were more sensitive to As_2_S_2_ in comparison to MDA-MB-231. Additionally, As_2_S_2_ affected cell morphology and triggered cell cycle arrest; in G_0_/G_1_ and G_2_/M phases in MCF-7 cells and G_2_/M and S phases in MDA-MB-231 cells. In both cell lines, As_2_S_2_ regulated cyclins (A2, B1, and D1) time- and dose-dependently. As_2_S_2_-induced apoptosis was associated also with increased proapoptotic p53 and decreased antiapoptotic Bcl-2 and Mcl-1 protein expression. Additionally, As_2_S_2_ downregulated the expression of PI3K and Akt. The blocking PI3K/Akt signals following As_2_S_2_ exposure in cancer cells could contribute to both induction of apoptosis and inhibition of cell viability [39].

As_2_S_2_ antiproliferative activity in MCF-7 cells was shown in both 2D- and 3D-culture systems with 3D spheroids less sensitive to arsenic in comparison to 2D cultured cells [55]. In another study, in both MCF-7 monolayers and spheroids, As_2_S_2_ at low concentrations, used together with an inhibitor of glutathione synthesis (BSO), exerted stronger anticancer action than without BSO [55]. Simultaneous use of As_2_S_2_ and BSO led to intensification of apoptosis and cycle cell arrest. These effects were mediated by changes in various proteins' regulation, among other synergistic inhibitory effect on PI3K/Akt signals. All these mechanisms could reverse resistance to arsenic in breast cancer [56].

Kim et al. demonstrated also anticancer effects of arsenic hexoxide (As_4_O_6_; AS6) used at a small, nontoxic dose of 5 *μ*M. Authors showed a significant attenuation of cell growth by AS6 in MCF-7 cells and assumed that in advanced breast cancer tumor necrosis factor *α* (TNF-*α*) is included in cancer progression and metastases [26]. TNF-*α* can induce apoptotic cell death, but yet many cancer cells are resistant to this action due to activating NF*κ*B-regulated gene products or NF*κ*B-mediated cellular processes. Thus, NF*κ*B inhibition should lead to enhanced TNF-*α*-induced apoptosis. Such NF*κ*B suppression, associated with decreased expression of cyclin D1, c-Myc, and also COX-2 in MCF-7 cells, was obtained using As_4_O_6_. Simultaneously, AS6 enhanced TNF-*α*-induced apoptosis [26].

In a more recent extended study, the same authors compared arsenic-induced changes in cytotoxicity and gene expression in MCF-7 and HUMEC (human mammary epithelial normal cells) and showed that AS6 at concentrations below 1 *μ*M had cytotoxic action stronger in cancer cells comparing to HUMEC [57]. The authors also showed different expression of genes regulating the cell cycle in HUMEC and MCF-7 cells. In cancer cells, expression of cyclin B1 was lower, but cyclin D1 was higher than in HUMEC, indicating disturbances in G_2_-M and G_1_-S transition in MCF-7 cells. Moreover, MCF-7 cells displayed increased expression of p21 mRNA. As p21 is one of the inhibitory proteins that arrest cell cycle (G1, S, and G2), the authors concluded that in MCF-7 cells, arsenic hexoxide inhibits cell cycle at these phases. AS6 caused also the impairment of DNA repair and increased genomic instability [57]. Findings of AS6 impact on the genome wide-gene expression using the RNA-seq analysis showed significant changes in expression of more than 7 thousand genes in MCF7 cells, about 6 times more than in HUMEC. Another difference was increased apoptosis, membrane transport, and response to hypoxia and endoplasmatic reticulum stress in MCF-7 cells in comparison to HUMEC. Furthermore, authors observed increased expression of *HSP70* and *HSP90* genes in MCF-7 cells. This observation could lead to a conclusion that exposure to AS6 enhances cellular stresses in breast cancer cells [57].

### 3.6. Arsenic Antiangiogenic Activity

An important anticancer mechanism of arsenic action may be its antiangiogenic activity [58]. In study performed by Jiang et al., ATO-induced changes in expression and secretion of VEGF were associated with impaired TNBC cells angiogenic ability. The latter was mediated by inhibition of interaction between enhancer of zeste homolog 2 (EZH2) and p65, downregulation of nuclear factor-*κ*B (NF-*κ*B) activity, and thus regulation of IL-6/Stat3 signaling pathway [58]. Attenuation of NF-*κ*B signaling pathway by ATO was shown also in a study from Nasrollahzadeh et al. This ATO effect was synergistically potentiated by BIBR1532, an inhibitor of hTERT, leading to attenuation of breast cancer cell proliferation [59].

## 4. Discussion

In a human observational study, Smith et al. showed that between 1958 and 1970, mortality from breast cancer was approximately two times lower in regions with extremely high concentrations of arsenic in water comparing to regions with low arsenic water concentrations.

In studies performed *in vitro* on human breast cancer cell lines, ATO induced apoptosis at concentrations of 1-2 *μ*M after 72 h. The authors estimated that similar arsenic concentrations could be found in people with high exposure to inorganic arsenic [60]. These ATO concentrations are considered safe, neither toxic nor carcinogenic, and the results of most presented *in vitro* studies have shown ATO anticancer activity in breast cancer cells for concentrations lower than 5 *μ*M. In some studies, ATO at concentrations higher than 5 *μ*M has been used. These studies concerned ATO nanoparticles, or ATO combination with another natural or chemical compound, or ATO used together with radiation or after transfection of cells with specific RNA. Other arsenic compounds (arsenic hexoxide, arsenic disulfide, sodium arsenite, MMA, or DMA), toxins weaker than ATO, have been used at concentrations higher than 5 *μ*M.

However, even at low doses, arsenic could affect the balance of intracellular processes towards pro- or anticancer effects, depending on breast cancer cell type, time of exposure and many extracellular and intracellular factors [61, 62]. Every *in vitro* study presented in this manuscript provides fragmentary knowledge about a selected process determining arsenic activity in breast cancer cells. Nevertheless, it remains mostly unclear which factors underlie molecular mechanisms of carcinogenic versus anticancer activity of arsenic. It is believed that an important factor is the activity of the apoptotic pathway, which promotes cell death and tumor growth inhibition. Since arsenic may induce and/or enhance apoptosis in breast cancer cells, it may have anticancer potential. On the other hand, apoptosis also appears to be the most common mechanism responsible for cancer cells resistance to chemotherapeutic agents or radiation therapy [63].

Arsenic-induced apoptosis and cycle cell arrest is associated with increased generation of intracellular ROS [44, 57, 64]. It is well known that ROS plays critical role in modulating oxidative stress. Because of arsenic prooxidative action, many studies concern antioxidant expression in “natural” and “transfected” malignant cells exposed to arsenic. In the MCF-7 CAT3 clone overexpressing catalase, both glutathione peroxidase and peroxiredoxin II protein levels and activity were decreased, while loss of MCF-7 CAT3 cells mobility and significantly decreased cell proliferation were observed. However, the clone of cancer cells remained sensitive to toxic effects of ATO. Interestingly, a significant increase in cell death rate was observed when MCF-7 CAT3 cells were treated with ATO at a high, toxic dose of 10 *μ*M [23].

Studies using TNBC cells transfected with FEN1-siRNA or pretreated with NAC, as well as studies on detection of intracellular GSH changes or expression and nuclear localization of Nrf2, provide grounds for a hypothesis concerning causes of significantly reduced GSH levels observed in cells treated with ATO plus knockdown of FEN1 [44]. Reduced level of GSH may be a consequence of GSH depletion and reduced Nrf2 nuclear transportation. The reduced GSH levels, and, in consequence, increased intracellular ROS, lead to increased apoptosis and DNA damage. Decreased expression of FEN1 could therefore enhance the anticancer effect of ROS inducer ATO [44]. The modification of intracellular oxidative status using transfected breast cancer cells should be studied further.

Results from most laboratory studies point to intracellular ROS generation by ATO and common cellular targets for arsenic anticancer effects. Mitochondria are intracellular structures especially sensitive to increased ROS generation. In breast cancer cells, oxidative stress damages mitochondrial integrity, causing mitochondrial dysfunction and cell death. These processes are associated with an increased expression of proapoptotic Bax, decreased expression of antiapoptotic proteins survivin and Bcl-2, and increased expression of cleaved PARP and caspases [41, 46, 49]. There is a dynamic relationship between mitochondrial function and activity of microRNA (miRNA), a small, noncoding RNA molecule that regulates gene expression at the posttranscriptional level. In breast cancer cells, ATO regulates activity of linear microRNAs such as miR-27a [51] or let-7a [52], whereas sodium arsenite upregulates expression of the newly recognized, noncoding circular RNA (circDHX34) in a dose-dependent manner [62]. Both arsenic compounds upregulate expression levels of antiapoptotic genes *BCL2* and *BCL2L1* and downregulate expression levels of proapoptotic genes *CASP8* and *CASP9*, promoting apoptosis in breast cancer cells.

Molecular targets for ROS generation and arsenic anticancer effects could be molecules which are indicators of apoptosis, as well as basal regulators of cell cycle and proteins responsible for DNA damage and activation of DNA damage repair systems. These targets could be clinically useful in the assessment of breast cancer risk and in the monitoring of arsenic anticancer effects. Humans with genetic polymorphisms associated with reduced isomerase *PIN1* expression are at a lower risk of cancer, whereas *PIN1* overexpression is associated with increased risk of proliferative diseases [42]. Another novel breast cancer biomarkers could be microRNAs, such as miR-27a, serving as a marker of poor prognosis, or let-7a, present in circulation, where its levels correspond with estrogen receptor status and lymph node status [51, 52]. The introduction of circDHX34 as a biomarker of exposure to sodium arsenite requires further research [62].

In breast cancer cells, ATO may be included in Notch-1, PIN1, and kinases oncogenic signaling pathways. The enhanced sensitivity of MCF-7 cells to ATO at lower concentrations may result from the inhibition of phosphatidylinositol-3 kinases (PI3Ks). PI3Ks are important intracellular secondary messengers involved in many cellular processes. The signaling pathway of PI3Ks and its nuclear effectors, in particular FOXO transcription factors, specifically target gene expression important in cell cycle arrest, cell death, ROS detoxification, DNA repair, energy homeostasis, and glucose metabolism. The PI3K inhibitor (BKM120) augmented ATO-induced antiproliferative effects by inducing G1 arrest and reducing the incorporation of bromodeoxyuridine into the synthesized DNA [46]. The latter was associated with suppression of human telomerase reverse transcriptase (hTERT) expression. Moreover, in the presence of the BKM120, ATO-induced apoptosis was increased [46]. Cell cycles G1, S, G2, and M phases play crucial role in cell differentiation, proliferation, apoptosis, and DNA damage repair [64]. ATO inhibited cell proliferation and induced G2/M cell cycle arrest and caspase-dependent death in MCF7 cells even without causing mitochondrial membrane disruption [65].

Based on aforementioned studies, currently, the greatest unsolved problem limiting arsenic potential as an anticancer therapeutic agent are arsenic toxic effects. Use of arsenic at low and relatively safe for human doses in studies *in vitro* and *in vivo* has not provided sufficient cytostatic effects. Some methods of combined therapy using arsenic plus another plant-derived or chemical agent provided promising results. The combination of ATO with all-trans retinoic acid has become a standard regimen for APL treatment [30, 32]. In various lines of breast cancer cells, cotreatment with arsenic and some natural products such as all-trans retinoic acid [42], tetrandrine [43], cryptotanshinone [41], epigallocatechin-3-gallate [49], or melatonin [48] inhibited cell growth and survival, as well as induced cell cycle arrest and apoptosis. Melatonin seems to be a particularly useful companion agent. The interaction between arsenic and melatonin was found to impact multiple neoplastic processes. In women with breast cancer melatonin proved to be a beneficial enhancer of ATO therapeutic effect, presumably by targeting ROS/Redd1 pathways [44, 48] with impact on the p53-dependent DNA damage response and p63-mediated regulation of epithelial differentiation gene [66]. Also, silymarin might be considered as an add-on to arsenic anticancer therapy, in order to prevent its systemic toxicity [50].

Another way to reduce arsenic toxicity would be to replace the most commonly used ATO with other, less toxic arsenic compounds [43, 55, 67]. Compared with ATO, arsenic disulfide is less toxic and has similar anticancer effects [39]. Arsenic hexoxide used at safe doses has diverse effects on cell proliferation and genome-wide gene expression in human normal and cancer cells, with a more pronounced activity in cancer cells [26, 57]. Promising research related to this issue concerns ATO nanoparticles (AsNPs). Subastri et al. demonstrated that in breast cancer cells, AsNPs exhibited a similar anticancer efficacy and significantly lower cytotoxicity compared to ATO [67].

Nanoparticles are also used to deliver arsenic to cells. This delivery should be appropriate with respect to time and dynamic of arsenic release, intracellular target sites, and arsenic concentration. The encapsulation of ATO in liposomes enhanced the therapeutic index of arsenic by reducing its side effects and increasing its concentration in tumors [68]. Also, human serum albumin and bovine serum albumin seem to be suitable candidates for arsenic transfer and delivery to cellular targets. Studies performed on breast cancer cell lines have shown a slow release of ATO after initial ejection from the microspheres, with a cumulative release close to 95% [64]. Polymersomes, nanoparticles made up of block copolymers arranged in a bilayer, surrounded by an aqueous core with the hydrophobic polymer wall, are used to deliver numerous chemotherapeutics [69]. Recently, stimulus-responsive drug delivery systems for controlled release of arsenic at target sites have been described. One example are mesoporous silica nanoparticles (MSNs) coated with polyacrylic acid (PAA) and pH-sensitive lipid (PSL) for synergistic delivery and dual-pH-responsive sequential release of arsenic trioxide (ATO) and another drug such as paclitaxel [70].

In addition to selection of an appropriate arsenic carrier, another problem potentially limiting use of treatment with ATO that requires consideration is tumor resistance to ATO cytotoxic effects. As development of a phenotype resistant to oxidative and metabolic stress may be mediated by arsenic-induced translationally controlled tumor protein (TCTP), improved outcomes in cancer treatment could be obtained by using TCTP inhibitors [37]. Another mechanism of breast cancer resistance to ATO is associated with the disruption of Pin1 binding to ATO. Promising study on mice showed not only synergistic anticancer effect of arsenic and ATRA, but also synergistic reduction of resistance to pharmacotherapy [42]. Also, synergistic inhibitory effect of arsenic and BSO on PI3K/Akt signals could reverse resistance to arsenic in breast cancer cells [56]. In epithelial breast cancer cells, mitochondrial functions are involved not only in anticancer effects but also in anticancer drugs resistance. It was found that a loss of stromal caveolin-1 (cav-1) expression is a predictive biomarker for poor clinical outcomes and tamoxifen resistance [71]. Cav-1 downregulation was observed in cancer-associated fibroblasts (CAFs), obtained as result of a coculture of normal fibroblasts and MCF-7 cells. CAFs led to intensified catabolic processes and significant impairment in mitochondrial function. Simultaneously, CAFs were able to induce antiestrogen resistance in breast cancer cells. It was demonstrated that mitochondrial activity drives tamoxifen resistance in MCF-7 cells, whereas mitochondrial inhibition, for instance by ATO, can resensitize cancer cells to tamoxifen [71].

Among goals of breast cancer treatment is prevention of metastases. Arsenic may be a factor inhibiting metastases by inhibiting angiogenesis. Apart from the arsenic-induced VEGF pathway attenuation [58] and Notch-1 pathway blockade [59], arsenic may downregulate nuclear factor-*κ*B activity [53]. The VEGF and Notch-1 pathways have been identified as essential regulators of angiogenesis, whereas NF-kappa B regulates VEGF activity.

**Figure 4:**
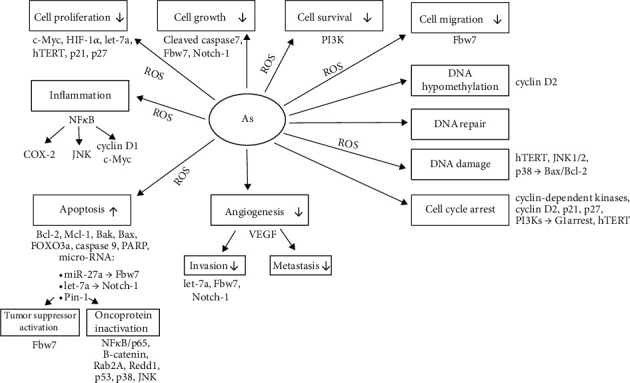
Mechanisms of arsenic effects in breast cancer cells based on *in vitro* studies.

To conclude, the literature of the last decade provides a lot of data on mechanisms behind anticancer effects of arsenic in breast cancer. Mechanisms of arsenic action unfold at various cell structural and functional levels. The main mechanism appears to include impact on intracellular oxidative status. Arsenic-induced increased generation of free radicals underlies inflammatory response and can trigger initiation and subsequent progression of cancer. However, the same pathway may promote apoptosis and cell cycle arrest, inhibiting cancer cell growth, viability, mobility, and proliferation (Figure 4).

Arsenic-induced changes at the level of membrane transport, cellular signaling pathways, as well as function of organelles such as mitochondria, endoplasmatic reticulum, and the nucleus at least in part determine arsenic carcinogenic or anticancer properties. The baseline activity of membrane receptors, cytokines, transcription factors, pro- and antiapoptotic enzymes as well as the cell cycle also affects cellular response to arsenic. The final cell response to arsenic depends on the stability of the genome as a whole, changes in the expression of many genes and the DNA repair ability. Beneficial effects of arsenic use are possible due to significant metabolic differences between cancer and healthy cells.

## 5. Directions for Further Research

Further efforts are needed in order to establish modes and doses of treatment with arsenic that would provide anticancer activity with minimal toxicity. Although arsenic compounds show promising anticancer activity *in vitro*, their clinical use in the treatment of breast cancer is currently limited. Arsenic cytotoxicity (Figure 1), increased risk of cancer and development of neurological, gastrointestinal, cardiovascular, and metabolic diseases as well as low arsenic solubility and rapid renal clearance are factors potentially limiting its therapeutic use. The risk of side effects of arsenic treatment increases in a dose- and time-dependent manner. The challenge is to determine a method of treatment with arsenic that would ensure its targeted delivery and effective concentration in cancer cells with minimal risk of systemic toxic effects.

Nanomedicine offers methods to obtain arsenic compound nanoparticles as well as nanoparticles of arsenic carriers to intracellular target sites. Use of arsenic nanoparticles requires in-depth research due to the high affinity of this arsenic form to the DNA. A further development of delivery systems of arsenic in combination with additional agents to cancer cells is needed. Use of add-on cytotoxic agents should simultaneously offer synergistic anticancer effects, allowing lower arsenic effective doses as well as antagonistic effects in relation to potential arsenic toxicity, with the benefit of both safer and more efficient cancer treatment.

## Figures and Tables

**Table 1:** Anticancer properties of arsenic (As) compounds in experiments *in vitro.*

As compound/concentration/time	Agents used in the combination with As compound	Breast cancer cell line	Anticancer effect of arsenic	Authors
Sodium arsenite (NaAsO_2_) 0, 3, 6, and 9 *μ*M for 72 h	Transfection with siRNA against circDHX34	MDA-MB-231	Sodium arsenite mediated upregulation of circDHX34 promotes apoptosis in hormone-independent breast cancer cells by regulating apoptotic genes	Li et al. [62]
Arsenic trioxide (As_2_O_3_: ATO) ATO : PTX ratio 1 : 2 and 2 : 1 for 24 h	Paclitaxel (PTX) mesoporous silica nanoparticles	MCF-7	ATO and PTX codelivered nanoparticles display a significant synergistic effect against MCF-7 cells, showing greater cell-cycle arrest in treated cells and more activation of apoptosis-related proteins than free drugs	Zhang et al. [70]
Arsenic hexoxide (AS_4_O_6_; AS6) 0, 0.1, 0.25, 0.5, 1, 2, 5, 10, 50, 100, and 200 *μ*M for 24 h or 48 h	None	MCF-7	AS6 could selectively arrest cell growth and induce cell death by modulating genome-wide gene expression, leading to compromised DNA repair and increased genome instability	Kim et al. [57]
Arsenic trioxide (ATO) 4, 5, 6, 7, 8, 9, 10, and 15 *μ*M for 24 h or 48 h	Epigallocatechin-3-gallate (EGCG) and gamma radiation	MCF-7	Synergistic antiproliferative effect of integrated therapy with green tea catechin, ATO, and gamma irradiation on MCF-7 cells	Changizi et al. [49]
Arsenic trioxide (ATO) 0, 2.5, 5, 6, and 10 *μ*M for 12, 24, or 48 h	Transfection with FEN1 siRNA	MDA-MB-231 MDA-MB-468	The combination of flap endonuclease 1 (FEN1) knockdown and ATO could induce apoptosis in TNBC cells death by promoting ROS generation	Xin et al. [44]
Arsenic trioxide (ATO) 0, 0.25,0.5, 1, 2, and 3 *μ*M for 48 h	BIBR 1532 (the human telomerase catalytic subunit- hTERT -inhibitor):	MCF-7 MDA-MB-231	The combination of ATO and BIBR1532 sensitized MCF7 and MDA-231 cells to lower concentrations of ATO, synergistically induced its antiproliferative effect in breast cancer cells by targeting the two key cancer-related pathways, hTERT and NF-*κ*B, and disrupting their feed-forward loop at the same time which result in the reduction of NF-*κ*B transcriptional activity and subsequent down-regulation of its target genes	Nasrollahzadeh et al. [59]
Arsenic trioxide (ATO) 0, 0 l5, 1, 2, and 3 *μ*M for 48 h	None	MDA-MB-231 Hs-578 T	ATO restrained the expression and secretion of vascular endothelial growth factor and impaired the angiogenic ability in TNBC cells	Jiang et al. [58]
Arsenic disulfide (As_2_S_2_) 0, 2, 4, 6, 8, and 12 *μ*M for 72 h	L-buthionine-(S,R) sulfoximine (BSO)	MCF-7 monolayers MCF-7 spheroids	BSO (a potent specific inhibitor of glutathione synthesis) in combination with As_2_S_2_ exerted potent anticancer synergism in both MCF-7 monolayers and spheroids	Zhao et al. [56]
Arsenic trioxide (ATO) 0, 2.5,3, and 5 *μ*M for 24 h or 48 h	BKM120 (orally bioavailable 2,6-dimorpholino pyrimidine deriva-tive, the selective small molecule inhibitors of PI3K)	MCF-7 MDA-MB-468	BKM120 sensitized MCF-7 cells to the lower concentrations of ATO. The significant anticancer effect of PI3K inhibition by BKM120 became even more evident when BKM120, either as a single agent or in combination with ATO, reduced clonogenic ability of anoikis-resistant stem-like MCF-7 cells. BKM120 augmented also ATO-induced antiproliferative effects through inducing G1 arrest and reducing the incorporation of bromodeoxyuridine into the synthesized DNA of drugs-treated cells, which was coupled with c-Myc-mediated suppression of hTERT expression	Alipour et al. [46]
ATO nanoparticles (AsNPs) 0.5, 1.0, 5.0, 10, and 15 *μ*g/ml)	None	MDA-MB-231 MCF-7	Antiproliferative activity of ATO nanoparticles (AsNPs) is coupled with binding to DNA without disturbing the structural integrity of DNA. AsNPs is less cytotoxic in comparison to ATO	Subastri et al. [67]
Arsenic trioxide (As_2_O_3_; ATO) 0, 0.125, 0.25, 0.5, 1, 1.5, and 2 *μ*M for 3 days	All-trans retinoic acid (ATRA)	MDA-MB-231	ATO targets Pin1 and cooperates with ATRA to exert potent anticancer activity. ATO inhibits and degrades Pin1 and suppresses its oncogenic function by noncovalent binding to Pin1's active site	Kozono et al. [42]
Arsenic disulfide (As_2_S_2_) 5, 10, and 15 M and 0, 4, 8, and 16 *μ*M for 24, 48, or 72 h	Ascorbic acid (AA) at L-buthionine-(S, R)-sulfoximine (BSO) at N-acetyl-L-cysteine (NAC)	MCF-7	As_2_S_2_ dose-dependently decreased the MCF-7 cell proliferation in both 2D- and 3D-culture systems. The 3D spheroids were less sensitive to As_2_S_2_ than the 2D cultured cells. Verapamil, an inhibitor of P-glycoprotein, partially enhanced the antiproliferative effects of arsenic	Uematsu et al., [55]
Arsenic disulfide (As_2_S_2_) 0, 4, 8, 12, 16, and 24 *μ*M for 24 h, 48 h, or 72 h	None	MCF-7 MDA-MB-231	Inhibition of cell viabilities, induction of apoptosis, and cell cycle arrest by regulating the expression of key proteins involved in related pathways with a dose- and time-dependent manner	Zhao et al. [39]
Sodium arsenite (NaAsO_2_, as ^III^) *in vitro*: 5, 10, 15, 20, 25, and 30 *μ*M for 48 h *in vivo*: 2 mg/kg/day for 10 weeks	Tetrandrine (Tetra)	MDA-MB-231 human breast cancer xenograft model	Intracellular cytotoxicity and antitumor activity of arsenic is enhanced by tetrandrine in a synergistic manner. The combined treatment upregulated the expression level of FOXO3a, and subsequently resulted in increase in the expression levels of p21, p27, and decrease of cycline D1, which occurred in parallel with G0/G1 phase arrest	Yuan et al. [43]
Arsenic trioxide (ATO) 0, 1, 2, 3, 4, and 5 *μ*M for 24, 48, or 72 h	None	MCF-7 MDA-MB-231 MDA-MB-468	Inhibiting a DNA methylation and induction of DNA hypomethylation by ATO is one of the molecular mechanisms underlying the ATO promoted cell cycle arrest. ATO via demethylation of the promoter-associated CpG islands resulting in upregulation of several cell cycle–related genes	Moghaddaskho et al. [21]
Arsenic trioxide (ATO) 0, 2, 4, 6, 8, and 10 *μ*M for 24 h or 72 h	Transfection with let-7a mimics or the nonspecific control	MCF-7 SK-BR-3	ATO upregulated let-7a level in breast cancer cells and by this way inibited cell growth, induced apoptosis and retarded cell migration and invasion	Shi et al. [52]
Arsenic trioxide (ATO) 0, 2, 4, 6, 8, 10, 12, and 14 *μ*M for 72 h	Transfection with miR-27a oligo-nucleotide or miR-27 mimics	MDA-MB-231 SK-BR-3	Inhibition miR-27a expression in breast cancer cells lead to suppression cell growth, migration, invasion, and trigged cell apoptosis whereas overexpression of miR-27a enhanced cell growth, motility, and inhibited apoptosis in breast cancer cells	Zhang et al. [51]
Arsenic trioxide (ATO) 0, 0.5, 1, 2, and 5 *μ*M for 48 h	Melatonin transfection with pcDNA3.1(+)-SOD and -catalase DNA transfection with siRNAs	MDA-MB-231 SK-BR-3	Melatonin enhances ATO-induced apoptotic cell death via sustained ROS mediated upregulation of Redd1 expression and the activation of the p38/JNK apoptotic pathway in human breast cancer cells	Yun et al. [48]
Arsenic trioxide (ATO) 0, 0.25, 1, 1.5, 4, and 6 *μ*M for 5 days	Cotylenin A	MCF-7 MDA-MB-231 T47D	Cotylenin A, a plant growth regulator and a potent inducer of differentiation in myeloid leukemia cells, significantly potentiated both ATO-induced inhibition of cell growth in a liquid culture, and ATO-induced inhibition of anchorage-independent growth in a semisolid culture in human breast cancer cells	Kasukabe et al. [47]
Sodium arsenite (NaAsO_2_) 200 *μ*M for 2 h	Quercetin Silymarin	MCF-7 ZR-75-1	Nitrosative stress may be an anticancer mechanism exerted by arsenic depending on the redox cellular response that could be modified by dietary antioxidants such as flavonoids	Soria et al. [50]
Inorganic arsenite (As) monomethylarsonous acid (MMA(III)) dimethylarsinous acid (DMA(III)) 1, 2, 5, 10, 20, 50, and 100 *μ*M for 24 h	Cryptotanshinone (CPT)	MCF-7	The combination of MMA(III) with CPT enhanced anticancer effects at low doses, connected with redistribution of proapoptosis related proteins Bax and Bak in the mitochondria, together with activation of poly(ADP-ribose) polymerase (PARP) and caspase-9	Zhang et al. [41]
Arsenic hexoxide (As_4_O_6_): 0, 0.1, 0.5, 1, 2, and 5 *μ*M for 1 h	None	MCF-7	As_4_S_6_ suppressed NF-*κ*B activation in both TNF-*α*-treated and control cells, and suppressed I*κ*B phosphorylation in a time-dependent manner, augmenting caspase-8 activation	Kim et al. [26]
Arsenic trioxide (ATO) 2, 4, 6, 8, 10, and 12 *μ*M for 72 h	None	MDA-MB-231 MCF-7 SKBR-3	ATO inhibited the Notch-1 and decreased the expression of Bcl-2 and NF-*κ*B resulting in cell growth and invasion inhibition and induction of apoptosis	Xia et al. [53]
Arsenic trioxide (ATO) 0, 1, 2, and 4 *μ*M for 6 days	Transfection with pERE-TATA-Luc+, rERa/pCI, and phRL-tk, nude mice xenograft model	MDA-MB-231	ATO reactivated ER*α* through competition with SAM for methylation of DNA and inhibition of DNMT1 protein along with partial dissociation of DNMT1 from the ER*α* promoter. ATO induced demethylation of the ER*α* promoter in ER-negative breast cancer cells was shown also in animal model	Du et al. [22]
Arsenic trioxide (ATO) 5 *μ*M for 24 h	Transfection with p21 or p27 shRNA	MCF-7	Change in the expression level of several genes that involved in cell cycle regulation, signal transduction, and apoptosis; increased the mRNA and protein levels of the cell cycle inhibitory proteins, p21 and p27	Wang et al. [54]
Arsenic trioxide (ATO) 2, 4, 8, 10, and 16 *μ*M for 24 h	None	MCF-7	ATO-induced apoptosis of MCF-7 cells associated with the activation of caspase-3 and decrease in HERG (potassium channels from the family of voltage-gated potassium channels)	Wang et al. [40]
Arsenic trioxide (ATO) 10 *μ*M for 48 h	Transfection with plasmid containing human catalase cDNA	Clone MCF-7 CAT3	Cells overexpressing catalase lost their ability to migrate, displayed a decrease of cell proliferation and were more sensitive to ATO used at high dose	Glorieux et al. [23]
Arsenic trioxide (ATO) 20 *μ*M for 24 h	Tamoxifen	MCF-7 Coculture of ER(+) MCF7 With fibroblasts	Mitochondrial activity in epithelial cancer cells drives tamoxifen resistance in breast cancer, whereas ATO, mitochondrial poison, is able to resensitize these cancer cells to tamoxifen	Martinez-Outschoorn et al. [71]
Arsenic trioxide (ATO) 0.5 *μ*M for 20 days 0, 0.5, 2, and 5 *μ*M for 24, 48, and 72 h	Transfection with plasmid shRNA	MDA-MB-231	A mild oxidative stress induced by low doses of ATO upregulates of translationally controlled tumor protein (TCTP) while a strong oxidative hit provided by ATO combined with glutathione depletion or condition of glucose deprivation causes a down-modulation of TCTP followed by cell death	Lucibello et al. [37]
Arsenic trioxide (ATO) 0.5, 1, 5, 10, 15, and 20 *μ*M for 24 to 72 h	Dichloroacetate (DCA)	T-47D MDA-MB-468 BT-20 MDA-MB-231	Reduction mitochondrial function through the inhibition of cytochrome c oxidase. The potentiation of ATO cytotoxicity by dichloroacetate is correlated with strong suppression of the expression of c-Myc and HIF-1a, and decreased expression of the survival protein Bcl-2	Sun et al. [45]

## Data Availability

Data are available on request.
